# Pivotal Role of Mitochondria in Macrophage Response to Bacterial Pathogens

**DOI:** 10.3389/fimmu.2019.02461

**Published:** 2019-10-23

**Authors:** Elodie Ramond, Anne Jamet, Mathieu Coureuil, Alain Charbit

**Affiliations:** ^1^Université de Paris, Paris, France; ^2^INSERM U1151, Institut Necker-Enfants Malades, Team 7, Pathogenesis of Systemic Infections, Paris, France; ^3^CNRS UMR 8253, Paris, France

**Keywords:** macrophage, immunometabolism, mitochondria, bacterial infection, cell polarization

## Abstract

Mitochondria are essential organelles that act as metabolic hubs and signaling platforms within the cell. Numerous mitochondrial functions, including energy metabolism, lipid synthesis, and autophagy regulation, are intimately linked to mitochondrial dynamics, which is shaped by ongoing fusion and fission events. Recently, several intracellular bacterial pathogens have been shown to modulate mitochondrial functions to maintain their replicative niche. Through selected examples of human bacterial pathogens, we will discuss how infection induces mitochondrial changes in infected macrophages, triggering modifications of the host metabolism that lead to important immunological reprogramming.

## Introduction

Macrophages play a unique role in phagocytosis and clearance of pathogens. This includes the secretion of anti-microbial effectors such as reactive oxygen species, nitric oxide, proton generation, anti-microbial peptides, and the establishment of a deleterious nutritive environment (1). In this context, bacteria have developed many tricks to subvert the intracellular environment and turn it beneficial for their own purpose, notably by altering mitochondrial integrity and function to influence energy generation, metabolism, and immune signaling.

**Figure 1:**
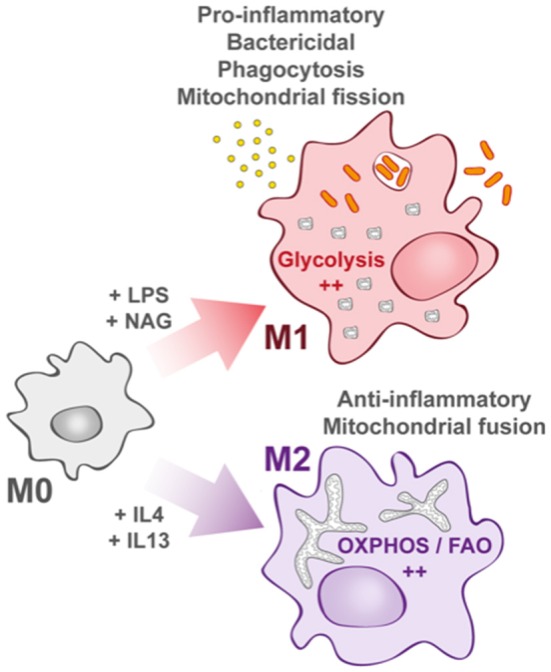
Macrophage activation upon bacterial infection. Quiescent macrophages (M0) have the ability to polarize into two antagonist cell types. At early steps of infection, M0 macrophages differentiate into M1 macrophages (upper part) that display a pro-inflammatory profile. They support highly efficient pathogen killing. This phenotype is associated with glycolysis induction. In contrast and later during infection, M0 macrophages can also differentiate into M2 macrophages (lower part). They rely on oxidative phosphorylation (OXPHOS) and fatty acid oxidation (FAO). They help in inflammation resolution.

Recent findings have linked the metabolic status of immune cells to the nature of immune responses against pathogens, leading to the concept of “immuno-metabolism” (2). In this review, we will first recall the specificities of macrophage metabolism and pathways that are activated upon bacterial invasion and discuss the metabolic reprogramming occurring upon pathogen infection. Then, we will describe how mitochondria play a key role in immune response and antibacterial effectors production. Finally, we will discuss examples of bacteria that either manage to shut down mitochondrial contribution in immune response or hijack mitochondrial metabolic activities for their own propagation. The concept of metabolic shift in response to environmental stresses, initially attributed to cancer cells in the mid-twentieth century, was extended to immune cells and specifically to macrophages. Indeed, macrophages have the capacity to change their metabolic profile with a remarkable plasticity depending on the environmental cues they receive. This is in accordance with the fact that monocytes and differentiated macrophages under high cellular turnover rapidly limit their proliferation to exclusively dedicate their energy to a robust immune response depending on pro-inflammatory signals (3). Upon immune challenge, macrophages switch from a quiescent or non-polarized state called “M0 macrophage” to two distinct activated states described as “classically activated” M1 macrophages or “alternatively activated” M2 macrophages (Figure 1), with the possibility to switch from M2 to M1 state (4, 5).

## Macrophage Activation Upon Bacterial Infection

Multiple pathogen associated molecular patterns (PAMPs) drive macrophage activation. This includes the detection of bacterial envelope components by cell surface receptors like the Toll-like receptors [TLR4, for lipopolysaccharide (LPS); TLR1, 2, and 6 for lipoproteins; and TLR5 (for flagellin)] or the cytosolic NOD-like receptors (NLRs) for peptidoglycan sensing. In addition to these well-known PAMPS, a broad family of other immune surveillance factors that detect and respond to these ligands have been very recently identified (6).

**Figure 2:**
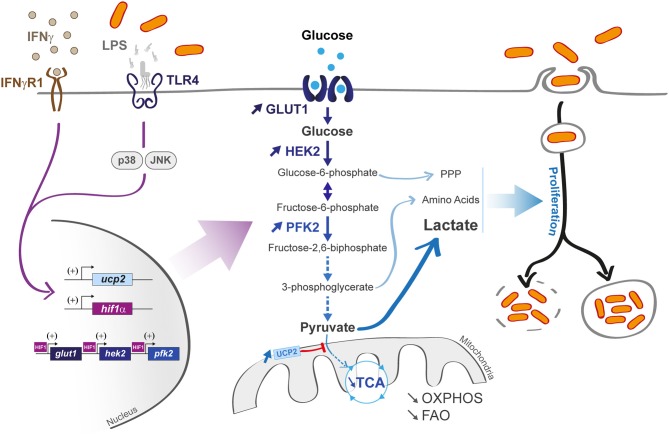
Macrophage infection affects host metabolic state and can induce an M1 profile. M1 macrophages are characterized by a hyper-activated glycolysis coupled with a limited mitochondrial activity (limited OXPHOS and FAO) upon lipopolysaccharide (LPS) or interferon gamma (IFNγ) activation (left part). The transporter Glut 1 and the glycolytic enzymes hexokinase 2 (HEK2) and phosphofrucotkinase 2 (PFK2) are activated (central part) in a hypoxia-inducible factor 1-α (HIF-1α)-dependent manner. LPS also activates the *uncoupling protein 2 (ucp2)* transcription via p38 and JNK pathways, leading to a decrease in pyruvate incorporation into TCA cycle. Some pathogens (vacuolar or cytosolic, right part) can use glycolytic derivatives [such as pentose phosphate pathway (PPP) metabolites, amino acids, and lactate] as nutrients to sustain their propagation.

M1 macrophages that differentiate under the influence of LPS and/or interferon-γ (IFN-γ) display a pro-inflammatory profile, coupled with a high phagocytic capacity (Figure 2). Notably, they support a glycolytic activity to rapidly produce adenosine triphosphate (ATP) and efficiently fuel the cell during acute inflammation at early stage of infection (5). The cell reprogramming toward an aerobic glycolysis is called “Warburg effect.” similarly to cancer cells reprogramming (7). This phenotype is explained by a LPS-activated hypoxia-inducible factor 1 α (HIF-1α). TLR4-dependent LPS activation was shown to increase HIF-1α gene transcription as well as HIF-1α protein translation in alveolar macrophages and the THP1 human macrophage cell line, independently from hypoxic induction (8, 9). HIF-1α knockout mice showed a limited glycolytic activity in myeloid cells, coupled with a limited TNF-α production, arguing for a prominent role of HIF-1α during infection (10). HIF-1α has the ability to enhance glycolytic flux by increasing the transcription rate of the genes *glut 1* (implicated in glucose transport) (11) and *hexokinase 2* (implicated in glucose phosphorylation) (12). HIF-1α activation also leads to a 9 fold increase in phosphofructokinase-2 (PFK2) expression, a key enzyme in glucose metabolism, by catalyzing the conversion of fructose-6-phosphate into fructose 2,6-bisphosphate (phosphorylation based reaction) (13, 14). At the end of glycolysis, pyruvate is mainly metabolized into lactate (Figure 2) that would help to limit excessive inflammation (15). Altogether, accumulated evidences demonstrate that LPS triggers a shift from an OXPHOS-dependent ATP production (taking place in mitochondria) to a glycolytic ATP production (in the cytosol).

The recent literature has demonstrated that several intracellular bacteria have the ability to induce a M1 phenotype upon macrophage infection, to create a supportive metabolic environment. Among them, *Legionella pneumophila* enhances glycolytic pathway to promote host serine production via 3-phosphoglycerate (16). Similarly, *Mycobacterium tuberculosis* uses pyruvate-derived lactate to favor its own growth (17). The obligate intracellular bacterium *Chlamydia trachomatis* has also been shown to increase host glucose uptake. Siegl et al. specifically showed that the bacterium induces p53 degradation, a strong inhibitor of the glucose-6-P-dehydrogenase (G6PD), a rate-limiting enzyme in the pentose phosphate pathway (PPP) (18). Indeed, G6PD silencing is associated with a non-replicative *C. trachomatis* state (19).

If M1 macrophages are the first line of defense against pathogens, M2 macrophages play also an important role in long-term fighting process and the resolution phase. They differentiate under the influence of IL-4 and/or IL-13 cytokines and have an anti-inflammatory role with the secretion of IL-10 and TGF-β (20, 21). Notably, they have limited microbicidal activity. IL-4, IL-10, and IL-13 were demonstrated to promote STAT6 transcription complex, leading to the activation of peroxisome proliferator activating receptors (PPARs) and increasing the oxidation rate of OXPHOS and FAO (22, 23). M2 status is also promoted via AMPK activation. In contrast to M1 macrophages, pyruvate generated through glycolysis is metabolized by mitochondria.

M2 macrophages can also represent a favorable niche for bacterial replication, favoring chronic infections. This property would be due to not only their limited bactericidal activity but also a metabolic-rich environment (24). Among them, *Salmonella typhimurium* and *Brucella abortus* preferentially invade M2 macrophages. They both activate PPARδ transcription to promote macrophage glucose entry and sustain a fully available carbon source (25, 26).

## Mitochondria are Key Players of the Metabolic Switch

Mitochondria are double-membrane organelles with an outer membrane and a folded inner membrane where the components of the electron transport chain (ETC) are localized. The mitochondrial ETC is composed of successive complexes, namely, complex I (or NADH dehydrogenase), complex II (or succinate dehydrogenase), complex III (or cytochrome *bc*_1_ complex), and complex IV (or cytochrome *c* oxidase). Electrons are transferred all along the ETC via redox reactions, generating a proton gradient (ΔΨ_m_) at the interface of the inner membrane. This electrochemical gradient (proton motive force) activates the ATP synthase that leads to the conversion of adenosine diphosphate (ADP) into ATP via a phosphorylation reaction (27).

Mitochondria are considered as “the powerhouses of the cell” as they generate a relatively high amount of ATP from high-energy molecules through OXPHOS compared to glycolysis (36 ATP molecules per glucose vs. 2 ATP molecules per glucose) (28). This metabolic shift also enables an increased mitochondrial membrane potential, protecting cell from apoptosis (29). It subsequently activates pro-inflammatory reactive oxygen species production. To optimally exert their bactericidal activity, mitochondria are found closely associated with phagosomes. Indeed, phagosomes generate signals to recruit mitochondria by activating the TLR-assembly TRAF6–ECSIT complex (30). As another metabolic signature, mitochondrial clearance through mitophagy also appears to be crucial for macrophage activation. Mitophagy consists in a mitochondria-specific autophagy process that aims to eliminate dysfunctional mitochondria, either in a stressed context, either associated with a programmed mitochondrial clearance (31–33). LPS/IFN-γ stimulation leads to a decrease in mitochondrial mass compared to IL-4/IL-13-activated cells, suggesting that mitophagy is activated during M1 macrophage polarization (34). This activation is regulated by hypoxia, via HIF-1α, subsequently activating the target gene BNIP3L/NIX, a master regulator of mitophagy. In the same work, the authors also demonstrate that mitophagy promotes glycolytic reprogramming, with the up-regulation of many glycolytic genes.

One subfamily of mitochondrial proteins, comprising the uncoupling proteins (UCP), also plays a key role in reprogramming macrophages early during infection. Under resting conditions, the uncoupling protein 2 (UCP2) harbors a high transcription rate, leading to high glutamine and fatty acid oxidation (35). Upon LPS activation, macrophages downregulate UCP2 transcription via JNK and p38 pathways activation, which are both in favor of glucose oxidation. In contrast, fatty acids are spared from mitochondria (36, 37). To summarize, this switch from oxidative phosphorylation to glycolysis creates a suitable environment, providing glycolytic intermediates as carbon sources to bacteria (38).

## Bacteria Target Host Mitochondria

Many bacteria have been shown to modify host metabolism by disturbing mitochondrial homeostasis and function. These organelles display dynamic transition by altering their morphology between a hyperfused state characterized by elongated mitochondria that dispatch in the cytosol and a fragmented state characterized by round-shaped mitochondria closely associated with the nucleus. GTPase proteins that belong to the Dynamin family ensure mitochondrial membrane remodeling. They influence membrane constriction, scission, or fusion. Dynamin-related/-like protein 1 (Drp1) and Dynamin2 (Dnm2) regulate mitochondrial constriction and scission. Drp1 is recruited to the outer mitochondrial membrane and forms a ring to narrow mitochondria membrane. Then, Dnm2 is recruited to the constriction neck to terminate membrane scission. In contrast, elongation implicates the two homologs Mitofusin1 (Mfn1) and Mitofusin2 (Mfn2). In a first step, outer mitochondrial membranes (OMMs) gather with the help of Mfn1 and Mfn2, leading to Mfns conformational change and switch to docking function. Following OMM fusion, the protein OPA1 and cardiolipins allow for inner mitochondrial membrane (IMM) fusion (39, 40). Recent findings show that modifying fusion or fission mitochondrial dynamics deregulates macrophage metabolism (41). *In vitro* analyses suggest that LPS-stimulated macrophages harbor shortened (fission profile) mitochondria via the activation of Drp1 and ROS production. In contrast, AMPK-activated macrophages show elongated (fusion profile) mitochondria (42). Fragmented mitochondria (fission process) are associated with a glycolytic metabolic profile, whereas elongated mitochondria (fusion process) rely on oxidative phosphorylation (43, 44). The fission process is important to limit the connectedness of the mitochondrial network. This mechanism is of importance in stress conditions as it reduces Ca^2+^ uptake efficiency from endoplasmic reticulum, a key effector in apoptotic signaling, and limits Ca^2+^ propagation and accumulation in the whole mitochondrial matrix, thus inhibiting cell death.

Hence, mitochondria play a critical role at all stages of infection by shaping macrophage metabolism. Remarkably, some pathogens have the ability to block this transition by modifying the mitochondria architecture. We will briefly review below the elucidated mechanisms developed by pathogens to counteract host immune responses. The bacteria described in this paragraph, with their mode of action, are listed in Table 1.

**Table 1:** Intracellular and extracellular pathogens that impact mitochondrial dynamics.

	**Processes affected in the infected host cell**	**Proteins implicated in mitochondrial alteration**
**INTRACELLULAR BACTERIA**		
**Vacuolar bacteria**		
*Salmonella typhimurium* (F)	• Decreases mROS production by targeting TRAF6 • Limits NLRP3 inflammasome activation	• SopB
*Legionella pneumophila* (F)	• Induces mitochondria fragmentation	• MitF, PitF/LegG1
*Chlamydia trichomatis* (O)	• Induces mitochondria elongation	
**Cytosolic bacteria**		
*Listeria monocytogenes* (F)	• Induces mitochondria elongation • Triggers NLRP3 inflammasome	• LLO
*Staphylococcus aureus*(F)	• Activates NLRP3 inflammasome to readdress mitochondria localization and function	• Alpha toxin
**EXTRACELLULAR BACTERIA**		
*Neisseria meningitidis*	• Induces cytochrome C release and apoptosis	• PorB
Enterohemorrhagic *Escherichia coli*	• Induces cytochrome C release and apoptosis	• EHEC hemolysin
*Helicobacter pylori*	• Induces cytochrome C release and apoptosis	• VacA
*Vibrio cholerae*	• Induces mitochondria fragmentation	• VopE

*(O) stands for “obligate” intracellular pathogens and (F) stands for “facultative” intracellular pathogens*.

### Disturbing Fusion–Fission Mitochondrial Dynamics and Mitophagy

Accumulating evidences suggest that bacteria can manipulate the mitochondrial network to favor their own replication. For example, Escoll et al. recently demonstrated that *L. pneumophila* alters mitochondrial dynamics by injecting into the cytosol effectors via its type 4 secretion system (T4SS). In particular, the *L. pneumophila* T4SS effector MitF (also called LegG1) was shown to induce a Drp1-dependent mitochondrial fragmentation, promoting a “Warburg-like” effect, already at 6 h post-infection. This cell reprogramming leads to a switch from mitochondrial respiration to glycolytic oxidation, which creates a favorable niche (45). In parallel to this mechanism, it was also shown that *L. pneumophila* produces a serine protease effector named Lpg1137 that degrades syntaxin 17 (Stx17), a soluble *N*-ethylmaleimide-sensitive factor attachment protein receptor (SNARE) protein. Stx17 promotes mitochondrial fusion (46). *Listeria monocytogenes* also developed tricks to subvert host mitochondrial network. Upon infection, bacteria induce a rapid mitochondrial fragmentation (within 1 h) in epithelial cells, caused only by the precocious release of extracellular listeriolysin O (LLO). By promoting mitochondria fission, LLO induces mitochondrial membrane potential loss, leading to dropped ATP production (47, 48). In the case of *L. monocytogenes*, the induction of a mitochondrial fission state needs to be induced upon bacterial entry and must be transient to favor optimal bacterial proliferation. Notably, in this context, mitochondrial fission is not associated with cell death, in contrast to most fragmentation processes that are primed by pathogens. It is supposed that the bacteria promote mitochondrial fission to avoid Ca^2+^ accumulation to cytotoxic levels that would lead to cell death. The authors also propose that it could be a way to affect macrophage bioenergetics status and subsequently cell immune function. It is important to note that the mitochondrial fission state (induced by using siRNA approach to silence Mitofusin 1 and/or Mitofusin 2) has to be time-limited, as it restricts intracellular bacterial proliferation (47). The obligate intracellular pathogens *Chlamydia* spp. is also able to modify mitochondrial morphology. Early during infection course, *Chlamydia psittaci* is closely associated to mitochondria organelles (49). *C. trachomatis* induces the host miRNA miR-30c-5p overexpression that directly down-regulates *drp1* gene expression, blocking mitochondrial fragmentation and promoting mitochondrial oxidative phosphorylation and ATP synthesis (50). However, at later time points (12 h post-infection), *C. trachomatis* relies on its own respiratory metabolism based on a sodium gradient to produce energy at the expense of mitochondrial ATP generation (51). Extracellular pathogens can also induce mitochondrial fission. For example, *Vibrio cholera* was shown to inject its T3SS effector protein VopE into the host cell. The VopE protein then binds to mitochondrial Rho GTPases Miro1 and Miro2 and stimulates their GTPase activity, preventing Mfn1-induced mitochondria fusion (52).

Intracellular pathogens can also induce programmed cell death dedicated to mitochondria. As a first proof of concept, it was shown that *Salmonella-*infected macrophages display autophagosomes that contain mitochondrial membrane structures, a mechanism dependent of the type III secretion system protein SipB. Immunofluorescence experiments showed colocalization in autophagic vesicles between mitochondrial markers and SipB (53). A very recent work also pointed out the importance of mitophagy during *L. monocytogenes* intracellular infection at early time points. It was shown that *L. monocytogenes* induces mitophagy through the virulence factor LLO. LLO induces NLRX1 activation, a Nod-like receptor containing a LC3-interacting region that promotes autophagic degradation (54).

### Destabilizing the Host Cell ETC

Targeting the ETC by inducing cytochrome C solubilization is another way to alter macrophage energy generation and induce apoptosis. Cytochrome C plays two roles in the cell: (i) on one hand, it acts as one intermediate of the ETC and supports the electron diffusion; (ii) on the other hand, when released into the cytosol, it binds to APAF-1 and pro-caspase 9, forming an apoptosome complex. This initiates the maturation of pro-caspase 9 into caspase 9 and starts the apoptotic process with the subsequent maturation of the effector caspases 3, 6, and 7 (55).

The extracellular pathogen *Neisseria meningitidis* was shown to target cytochrome C (56). During infection, *N. meningitidis* secretes outer membrane vesicles (OMVs) that enter macrophages, reach mitochondria, and release the PorB porin. PorB induces a loss of mitochondrial membrane potential, associated with cytochrome C release and macrophage apoptosis (56, 57). Another extracellular pathogen, the enterohemorrhagic *Escherichia coli*, also acts on mitochondria integrity. The bacterium expresses an hemolysin that is addressed to host mitochondria via OMVs, inducing cytochrome C solubilization and later on, cell apoptosis (58). A third example of extracellular pathogen acting on mitochondria integrity is *Helicobacter pylori*. This bacterium, upon infection, secretes a toxin named VacA that enters the host cell and targets mitochondria. As for the two former extra-cellular pathogens, it permeabilizes the IMM, leading to cytochrome C release and subsequently to cell death (59). Of note, suggesting that this mechanism is not specific to extracellular bacteria, Abarca-Rojano et al. showed that virulent *Mycobacterium tuberculosis* H37Rv strain induced a drop in ΔΨ_m_, by disturbing the mitochondrial external membrane integrity, consequently leading to a release in cytochrome C (60).

### Blocking mROS Generation

Mitochondrial ROS (mROS) play a critical role as anti-microbial molecules in M1 macrophages and contribute to pathogen clearance during infection. Indeed, since H_2_*O*_2_ and ${\text{O}}_{2}^{∙-}$ are volatile molecules, they can readily cross membranes (61) and act directly on pathogens. Many pathogens are able to limit phagosomal NADPH oxidase and NOX2 functions directly in the phagosome. *S. typhimurium* causes severe intestinal infections that may lead to death. To prevent mROS production that would be deleterious for its own survival and proliferation, *S. typhimurium* has developed mechanisms where it blocks mROS accumulation after 30 h of infection. Bacteria secrete the effector protein SopB, encoded by the Salmonella pathogenicity islands (SPI)-1 T3SS. SopB binds to the adaptor protein TRAF6 (already at 2 h of infection) and blocks its recruitment to the mitochondria while promoting its binding to the Salmonella-containing vacuoles (SCVs). This mechanism also allows a delayed host cell apoptosis and limits the production of pro-inflammatory cytokines (62).

### Modulating NLRP3 Inflammasome

*S. typhimurium* is able to delay NLRP3 activation to reinforce immune evasion, 12 to 16 h post-infection in bone marrow-derived macrophages (BMDMs). This involves the controlled production of TCA metabolites by Salmonella TCA cycle enzymes such as the isocitrate dehydrogenase and the aconitase (63). In contrast, some bacteria induce NLRP3 inflammasome activation to promote host cell invasion. For example, *Staphylococcus aureus* secretes an alpha toxin (AT, also known as alpha-hemolysin Hla) that promotes NLRP3 activation and consequently recruits mitochondria far from phagosomes. NLRP3-knockout mice or macrophages infected with *S. aureus* lacking AT increase their power in bacterial killing. Interestingly, in the absence of NLRP3, mitochondria localize with phagosomes and actively generate mROS that induce pathogen killing (64). *L. monocytogenes* also triggers NLRP3 inflammasome through LLO.

### Itaconate Detoxification

Itaconate is a macrophage-induced metabolite that acts as an antimicrobial effector (see below) that blocks bacteria glyoxylate pathway by inhibiting the isocitrate lyase and consequently bacterial growth (65). Interestingly, Sasikaran et al. showed that both *Yersinia pestis* and *Pseudomonas aeruginosa* encode an itaconate coenzyme A (CoA) transferase, an itaconyl-CoA hydratase, and a (*S*)-citramalyl-CoA lyase, three enzymes that synergistically convert itaconate into pyruvate and acetyl-coA. It is tempting to suggest that this conserved property is the result of convergent evolution to dampen the innate immune response (66).

## Mitochondria Trigger Macrophage Host Defense Mechanisms

**Figure 3:**
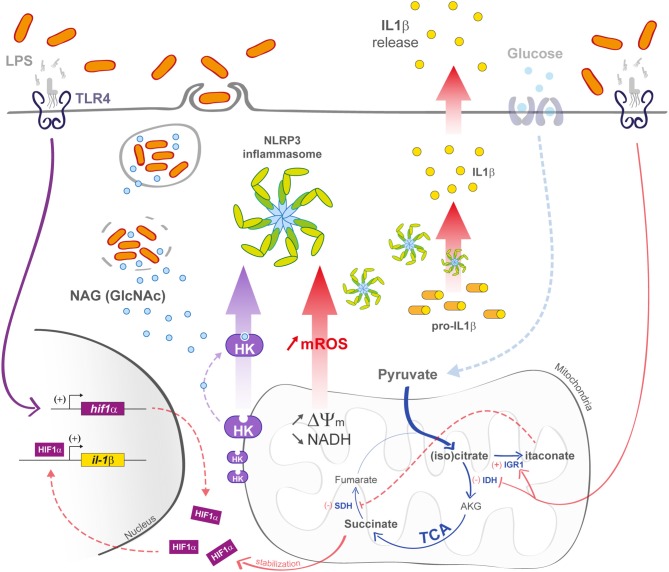
M1 macrophage defenses upon bacterial infection. In M1 macrophages, the mitochondrial TCA cycle is shunted at the isocitrate dehydrogenase (IDH) step, subsequently leading to itaconate formation. The succinate dehydrogenase (SDH) step is also arrested, leading to succinate accumulation and IL-1β transcription increase in a Hif1-α-dependent manner. Decrease in TCA cycle activity efficiency is also responsible for an increase in ΔΨ_m_ that induces the production of mROS. In bacteria-infected macrophages (left part), phagosomal maturation is responsible for N-acetylglucosamine (GlcNAc) release. GlcNAc binds mitochondrial hexokinase (HK) and induces its cytosolic release that activates NLRP3 inflammasome. mROS also induce NLRP3 activation.

Many processes that affect mitochondria drive an immune response in macrophages. In this part, we tried to summarize (Figure 3) (21, 67, 68).

### Metabolic Intermediates as Key Protectors

Among them, a combined metabolomic and transcriptomic approach identified several TCA cycle breakpoints induced upon LPS activation, promoting a protective phenotype against pathogens (69). Notably, Jha et al. identified a breakpoint at the isocitrate dehydrogenase step that leads to an accumulation of citrate and isocitrate. As a consequence, citrate is redirected to the cytosol where it is metabolized into acetyl-coA and oxaloacetate, providing a source of nicotinamide adenine dinucleotide phosphate (NADPH) for ROS and NO production (70). Interestingly, mutation in the gene encoding mitochondrial citrate carrier (a protein that exports citrate from mitochondria to cytosol) was shown to significantly reduce the amount of NO, ROS, and prostaglandin production upon LPS challenge (71).

In parallel, cis-aconitate leads to the production of the dicarboxilic acid itaconate by the mitochondria-associated enzyme immune responsive gene 1 (*Irg1*). Itaconate is produced within the first hours of infection (72), and it is the most abundant metabolite found in LPS-stimulated BMDMs as it reaches an 8 mM concentration in the cytosol (73). Interestingly, itaconate also protects activated macrophages from too pronounced pro-inflammatory state (72). Jha et al. also demonstrated the occurrence of a second breakpoint at the succinate dehydrogenase step that converts succinate to fumarate, leading to a significant increase in succinate concentration (69). In LPS-stimulated macrophages, succinate induces HIF-1α stabilization and activation, leading to an increase in IL-1β production via the activation of the HIF-1α responsive element (HRE) promoter, and consequently ending with a sustained pro-inflammatory state (74).

Similarly, it was demonstrated in zebrafish model that Irg1 (the mitochondria-associated enzyme immune responsive gene 1) links oxidative phosphorylation and mROS production. Indeed, it was shown that Irg1, upon infection with *S. typhimurium*, is expressed in macrophage through the glucocorticoid receptor and JAK/STAT signaling pathways and stimulates fatty acid consumption via OXPHOS, leading to mROS production and subsequently to bacterial killing (75).

### NLRP3 Inflammasome Activation

Another way to activate macrophage innate immunity is to stimulate inflammasome formation (Figure 3), and growing evidence suggests a contribution of mitochondria in the activation of these macromolecular complex platforms upon cellular infection (76). Notably, it was shown that N-acetylglucosamine (GlcNAc), a peptidoglycan sugar subunit from bacterial cell wall, induced the NOD-like receptor family pyrin domain-containing 3 (NLRP3) inflammasome formation in the cytosol. GlcNAc is released during the phagosomal step of bacterial infection and provokes hexokinase dissociation from the mitochondrial outer membrane. The hexokinase enzyme is involved in glucose phosphorylation at the very first step of the glycolysis and is closely related to the voltage-dependent anion channel (VDAC) in the outer mitochondrial membrane. After cell triggering by PGN, hexokinase dissociates from VDAC, causing NLRP3 activation (77). In parallel, decrease in cytosolic K^+^ also triggers NLRP3 inflammasome. Upon LPS stimulation, the P2X7 cation channel induces Na^+^ and Ca2^+^ import, which correlates with mROS production (78). Altogether, these mechanisms enhance NLRP3 inflammasome formation. In parallel, P2X7 activation also activates K^+^ export via the TWIK2 channel that is involved in potentiating mROS generation and concomitantly NLRP3 activation (79). Other reported mechanisms induce NLRP3 inflammasome activation. Subramanian et al. showed that upon stimulation by the bacterial toxin nigericin, or LPS, NLRP3 interacts with mitochondria via the mitochondria-associated adaptor protein MAVS, leading to ASC (Apoptosis-associated speck like protein) polymerization and downstream activation of caspase 1 and cytokine production (80). Assembly of the NLRP3 complex leads to the autocatalytic activation of caspase-1 and then the pro-inflammatory cytokines IL-1β and IL-18. Knockout mice for IL-1β and IL-18 show a burden in bacterial load associated leading to higher mortality upon infection (81).

### The Mitochondrial Uncoupling Protein UCP2 Modulates Macrophage Immune Response

UCP2 protein (described in the first part of this review) also plays a key role in immune response against pathogens (82, 83). Specifically, it was shown that UCP2, by controlling mitochondria-derived reactive oxygen species, is able to regulate macrophage activity and immune response but only when it is downregulated (83, 84). UCP2 transcription is down-regulated upon LPS stimulation, promoting the inducible form of the NO synthase, nitric oxide (NO), and ROS (36, 85). Indeed, UCP2-deficient macrophages are more prompt to clear *S. typhimurium* intracellular infection (83). Moreover, it was shown that *Ucp2(–/–)* mice have a better survival rate than *Ucp2(*+*/*+*)* mice against *L. monocytogenes*, which is surprisingly not due to a better macrophage clearance (analyzed in BMDM). Instead, the authors show that macrophages from *Ucp2(–/–)* mice secrete cytokines such as IFNγ, IL6, and IL1β, or IL10 that helps to recruit monocytes and neutrophils (86).

### TRAF6 as a Mediator of the Immune Response

Finally, it was also shown that, under LPS stimulation, tumor necrosis factor receptor-associated factor 6 (TRAF6) recruited the evolutionary conserved signaling intermediate in Toll pathways protein (ECSIT) to the outer mitochondrial membrane, leading to the activation of the (ETC) complex I. mROS can then rapidly spread in the whole cell and increase the mitogen-activated protein kinase (MAPK) activity and enhance the production of inflammatory cytokines such as IL-6, IL-10, and TNF-α (87).

### Mitochondria as Physical Obstacles

To finish, it was shown that mitochondria also play a “physical role” in containing intracellular pathogen in macrophage. *Shigella flexneri* infection in HeLa cells leads to the formation of cage-like structures, made of septins, around bacteria. Proteomic analyses of septin-associated proteins revealed that 21.4% are mitochondrial proteins. Indeed, infection induces mitochondria migration around the bacteria and mitochondrial fusion events through Drp1. Drp1 recruits septins (and septins simultaneously promote Drp1-mediated fission) that promote cage formation and bacterial autophagy via the phagophore assembly (88, 89).

## Concluding Remarks

We have shown here that intracellular bacterial pathogens are able to disturb and/or reprogram macrophages' metabolic health to their own profit in order to establish successful infections.

At this stage, many questions remain unanswered and issues regarding the experimental settings must be addressed. First, most of the classically used cellular models are derived from immortalized cell lines that have an altered metabolism, with an enhanced Warburg-like metabolism, as in the M1 macrophage subtype (7). In addition, the media used in *in vitro* analyses are supplied with substrate concentrations that are often significantly more important than in physiological *in vivo* compartments, which may introduce bias in their physiological relevance. Furthermore, data obtained in mouse cells cannot be systematically extrapolated to human cells. Indeed, it was recently shown that even if mouse and human macrophages (mBMDM and hBMDM) share many similar features, differences in metabolic reprogramming were observed between these two cell types. For example, Vijayan et al. very recently showed that hBMDM relied more on OXPHOS than on glycolysis metabolism upon LPS challenge (90).

From a clinical point of view, an interesting strategy would be to target mitochondria with drugs in order to limit infection and, more specifically, intracellular bacterial replication. Researches that focus on this field are currently still very limited. However, one interesting work from Jessop et al. showed that cyclosporine, which blocks calcium efflux from mitochondria and impairs the mitochondrial membrane permeability transition pore (mPTP), also limited *Francisella tularensis* sp. *tularensis* replication in mBMDM by reducing CFU counts in cells up to 100-fold (91).

It is also important to note that mitochondrial disorders can be responsible for bacterial infection susceptibility. Several clinical reports including cohorts of patients with primary mitochondrial diseases (that refers to abnormal oxidative phosphorylation) and presenting gut disorders, eye muscles paralysis, hearing impairment, and/or peripheral neuropathy were noted to have frequent infections. A large fraction of patients are infected with pathogens such as *S. aureus, P. aeruginosa, Clostridium difficile, E. coli*, and many others, associated with different infection sites such as respiratory tract, digestive tract, urinary tract, and more generally sepsis (92–94).

We have attempted to highlight the key role played by mitochondria in the tight interplay between host metabolic modifications and immune responses during a bacterial infection. It is now well-demonstrated that these organelles can satisfy cell requirements for energy demands and concomitantly prepare phagocytosis to sustain a resolution phase in macrophage. Consequently, modifying mitochondrial activity can modulate macrophage immune response, and targets, such as PPARγ, MAPK, or HIF1α, have been identified. Immunometabolism linked to microbiology is a new and rapidly evolving field that may bring new therapeutic strategies to fight infectious and inflammatory diseases.

## Author Contributions

ER, AJ, MC, and AC co-wrote the manuscript and designed the figures.

### Conflict of Interest

The authors declare that the research was conducted in the absence of any commercial or financial relationships that could be construed as a potential conflict of interest.
